# Stapling Through a Bougie During Sleeve Gastrectomy in a Superobese Patient—a Video Vignette

**DOI:** 10.1007/s11695-020-04790-z

**Published:** 2020-07-02

**Authors:** Christoph Bichler, Julia Jedamzik, Daniel M. Felsenreich, Felix B. Langer, Magdalena Eilenberg, Natalie Vock, Katharina Steinlechner, Jakob Eichelter, Lisa Gensthaler, Gerhard Prager

**Affiliations:** Division of General Surgery, Department of Surgery, Vienna Medical University, Waehringer Guertel 18-20, 1090 Vienna, Austria

**Keywords:** Sleeve gastrectomy, RYGB, Video vignette, Bougie

## Abstract

**Purpose:**

Bariatric-metabolic surgery in superobese patients (BMI > 50 kg/m^2^) is very challenging indeed with little room for error. In many cases, a two-step procedure is required, since more complex primary bariatric procedures can be technically demanding and bearing a relevant risk for the patient. At our institution, laparoscopic sleeve gastrectomy (SG) is the preferred primary procedure, followed by a conversion to either SADI-S or Roux-en-Y gastric bypass (RYGB) after initial weight loss is achieved [1, 2]. This video aims at demonstrating the conversion from primary SG to RYGB due to an adverse event in a 45-year-old superobese female patient (weight, 170 kg; BMI, 73 kg/m^2^).

**Methods:**

An intraoperative laparoscopic video has been anonymized and edited to demonstrate the course of the operation on the patient mentioned above.

**Results:**

The start of the procedure was uneventful. After a successful mobilization of the greater curvature, the stomach was resected with an electronic stapling device guided by a firm 36-french bougie (Rüsch, Germany) towards the angle of His. Due to a limited view, a stapler was placed over the bougie, which resulted in the stomach being subtotally transected, the staples attaching the bougie to the sleeve about 5 cm from the gastroesophageal junction. Salvage surgery after removing the remnants of the bougie was a conversion to RYGB.

**Conclusion:**

When performing a bariatric-metabolic surgery in superobese patients, an extended skill level is required to provide a solution, should anything go wrong. Therefore, we suggest bariatric-metabolic surgery in superobese patients to be performed solely and specifically at high-volume centres.

**Electronic supplementary material:**

The online version of this article (10.1007/s11695-020-04790-z) contains supplementary material, which is available to authorized users.

## Introduction

Bariatric-metabolic surgery in superobese patients (BMI > 50 kg/m^2^) is very challenging indeed with little room for error. In many cases, a two-step procedure is required, since more complex primary bariatric procedures can be technically demanding and bearing a relevant risk for the patient. At our institution, laparoscopic sleeve gastrectomy (SG) is the preferred primary procedure, followed by a conversion to either SADI-S or Roux-en-Y gastric bypass (RYGB) after initial weight loss is achieved [1, 2].

## Purpose

This video aims at demonstrating the conversion from primary SG to RYGB due to an adverse event in a 45-year-old superobese female patient (weight, 170 kg; BMI 73 kg/m^2^). In preparation, the patient received a dietary counselling and was able to lose 9 kg (EWL (excess weight loss), 8%; TWL (total weight loss), 5%) on a low-carbohydrate diet.

## Methods

An intraoperative laparoscopic video has been anonymized and edited to demonstrate the course of the operation on the patient mentioned above.

## Results

The start of the procedure was uneventful. After successful mobilization of the greater curvature, the stomach was resected with an electronic stapling device guided by a firm 36-french bougie (Rüsch, Germany) towards the angle of His. Due to a limited view, a stapler was placed over the bougie, which resulted in the stomach being subtotally transected, the staples attaching the bougie to the sleeve about 5 cm from the gastroesophageal junction. Salvage surgery after removing the remnants of the bougie was a conversion to RYGB. After surgery, the patient underwent an uneventful postoperative course.

## Discussion

The decision to change strategies intraoperatively was made for the following reasons. Suturing the defect was eliminated due to the risks of postoperative stenosis and early leaks. Thus, the decision was made to staple proximal to the area harmed. A conversion to RYGB was chosen based on the fact that RYGB is a low-pressure system (as opposed to SG) and is thus well suited to treat intra- and postoperative complications [3, 4]. Every bariatric patient should be informed preoperatively about a possible intraoperative conversion/change of strategy.

## Conclusion

When performing bariatric-metabolic surgery in superobese patients, an extended skill level is required to provide a solution, should anything go wrong. Therefore, we suggest bariatric-metabolic surgery in superobese patients to be performed solely and specifically at high-volume centres.

## Electronic Supplementary Material

ESM 1 (MP4 238800 kb)
